# The network structure of depressive symptomatology in Peruvian adults with arterial hypertension

**DOI:** 10.12688/f1000research.27422.1

**Published:** 2021-01-12

**Authors:** Cristian Ramos-Vera, Jonatan Banos-Chaparro, Roseline Oluwaseun Ogundokun

**Affiliations:** 1 Faculty of Health Sciences, Research Area, Cesar Vallejo University, 640 Del Parque Avenue, San Juan de Lurigancho, 15434, Peru; 2 Norbert Wiener University, Peru, 46970, Peru; 3 Department of Computer Science, Landmark University Omu Aran, Omu Aran, Kwara State, 251101, Nigeria

**Keywords:** Hypertension, Depression, Mental Health, Health Surveys, Peru

## Abstract

**Background:** Global arterial hypertension (HTA) has increased by 90% over the last four decades, and has increased by 1.6% in Peru over the previous four years. This study involved a network analysis of depressive symptomatology in Peruvian patients with HTA using network estimation.

**Method:** A representative cross-sectional study at the national level, using secondary data from 2019 Demographic and Family Health Survey (ENDES) was performed. The sample used included men and women of age over 17 years diagnosed with HTA and were able to respond to Patient Health Questionnaire-9 (PHQ-9).

**Results:** The symptoms of depressive mood (bridging force and centrality) and energy fatigue or loss (bridge centrality) play an essential role in the network structure, as does the feeling of uselessness in terms of closeness and intermediation.

**Conclusion:** The study highlighted the symptoms related to depressive mood and energy fatigue or loss as bridging symptoms, which could trigger a depressive episode in patients diagnosed with HTA.

## Introduction

Diagnoses of arterial hypertension (HTA) among other chronic non-communicable diseases are common ^1^. It necessarily requires a change of lifestyle that favors the adherence to pharmacological and psychological treatments, to reduce the development of cardiovascular diseases or psychological problems which complicates the patient’s health condition. An international study based on various surveys and reviews from 200 countries indicated that HTA cases worldwide have increased by 90% over the last four decades, with issues mostly identified in low- and middle-income countries ^2^. In Peru, the prevalence of HTA has increased in recent years: 2016 (8.6%), 2017 (8.7%), 2018 (9.5%) and 2019 (10.2%) ^3^. This increase is due to a rise in the population of older people and various lifestyle factors (such as food, minimal physical activity, alcohol consumption, among others) ^3^.

There is also evidence that patients with HTA have a higher incidence of emotional disorders, mainly depressive symptomatology (anxiety or stress), which interferes with their clinical treatment, leading to poor prognosis (not following the doctor’s instructions regarding medicines, minimal personal care) and preventing the acquisition of desirable behaviors to improve their quality of life ^4^. A recent study in the Peruvian population indicated that depressive symptoms are most likely to occur in the first year of diagnosis of hypertension ^5^. This reinforces the importance of considering evaluation by mental health professionals in improving primary care in persons diagnosed with HTA in Peru. 

Several studies have indicated that clinical interventions should primarily focus on depressive in patients with HTA ^4, 6^. Patients diagnose with HTA normally experience negative emotions due to the fact that they have to consume the drugs prescribed for treatment for the rest of their lives or for a very long period, these emotions are more powerful in situations where their condition is severe, and may generate feelings of loss of control or fear of failure, thus making it more likely that those with HTA condition can develop some emotional disturbances ^7^. These emotions are also related to the economic expenses involved in treatment (especially in low and middle-income countries) and the decrease in social interaction with friends or family ^1^. Ignoring negative emotions may result in physical disorders. These are likely to decrease adherence to treatments where psychological support is needed, especially the ones associated with risky behaviors such as alcohol consumption ^4, 8^.

Therefore, the research aimed to explore the network dynamics of depressive symptomatology in Peruvian adults with arterial hypertension from a network analysis approach, which allows a broad understanding of the interactions and the bridges of connection between the depressive symptoms and the study population.

## Methods

A secondary cross-sectional study was conducted based on data from ENDES 2019, which is a national representative survey that collects information on chronic non-communicable diseases and gives access to diagnostic and treatment services in Peru. ENDES design includes a two-stage random sampling technique, differentiated for rural and urban areas. In rural areas, the primary sampling units were groups of 500–2000 individuals and the secondary sampling units were the households within each of these groups. On the other hand, in urban areas, the sampling units consisted of blocks or groups of blocks with more than 2,000 individuals and an average of 140 households, and the secondary sampling units were the same as in rural settings from 36,760 sampled households, 34971 persons aged 15 and older were surveyed with the Health questionnaire. Details of data sampling, processing and collection are contained in the ENDES technical report produced by the National Institute of Statistics and Data Processing (INEI).

 Our sample included men and women over the age of 17 diagnosed with HTA. The diagnostic criteria for HTA were those with systolic blood pressure greater than and/or equal to 140 mmHg and/or a diastolic blood pressure greater than and/or equal to 90 mmHg ^9^, and had completed the Patient Health Questionnaire (PHQ-9) ^10^. Exclusion criteria were those who met the diagnostic criteria for hypertension, or did not report any blood pressure measurements, or who omitted any PHQ-9 questions. This allows evaluation of each of the nine DSM-IV depression criteria. PHQ-9 has four response options (0 = nothing at all, 1 = several days, 2 = more than half of days, and 3 = almost every day) and assesses the presence of depressive symptomatology in the last two weeks, the overall response score is in the range of 0 to 27.

### Sample

A total of 2915 participants were included in this study, of whom 1106 (37.94%) were male, and 1809 (63.06%) were female. The mean age was 57.9 years (standard deviation: 16.9), with 1456 (49.88%) being older adults (55 years old and over). Of these, 1144 (39.24%) had completed primary education, 844 (28.95%) secondary, 637 (21.75%) higher and 290 (9.94%) did not answer. Regarding the participants native language, 2106 (72.24%) indicated Spanish, 688 (23.6%) Quechua and 121 (4.16%) a different native language.

### Data analysis

For the analysis of the data, the graph package version 1.6.5 ^11^ was used in the statistical software R version 4.0.3, which allows estimation of a Gaussian chart model (GGM) of a regularized partial correlation network to model the interaction between the components of PHQ-9 as autonomous entities, which are represented as circles, called “nodes”. Nodes are connected by lines, called “borders.” Borders in GGM can be understood as conditional dependency relationships between elements. If two items are connected to the resulting network, they are dependent after all other items are adjusted. This analysis presents statistical coefficients of effect size (≤ 0,1 = small; > 0,1 to < 0,5 = moderate; ≥ 0,5 = large) to determine network connections. The precision of the edge weights was estimated to provide greater stability to the results, with a precision of 95% of the confidence intervals through Bootstrapping of 5000 samples around each edge in the network ^10^. Also considered were the most commonly used centrality indices in psychological networks: force, proximity, intermediation ^12^. 

### Ethical considerations

The investigation did not require the approval of an ethics committee because it only involved the analysis of secondary data obtained from a public and open source, which does not require the identification of the participants and maintains the anonymity of the participants.

## Results

In Table 1, the mean scores for each item of the PHQ-9 are shown ^13^. This shows that people with arterial hypertension have a higher level of “Depressed mood”. The item also reports that there is a more significant measure of force in terms of other depressive symptoms. Another essential item on the web was Tired or little energy. The elements of lower centrality were “Moving/restless” and “Appetite change”.

**Table 1.** Mean and power centrality of items of PHQ-9.

Depression symptoms (PHQ-9)	Mean (ME)	Strength
PH1. Interest loss	0,63	-0,60
PH2. Depressed mood	0,77	1,93
PH3. Trouble sleeping	0,63	-0,25
PH4. Tired or little energy	0,55	0,96
PH5. Appetite change	0,44	-1,06
PH6. Feelings of worthlessness	0,38	0,06
PH7. Trouble concentrating	0,40	0,27
PH8. Moving slowly/restless	0,23	-1,37
PH9. Suicidal thoughts	0,29	-0,47

**Figure 1.**
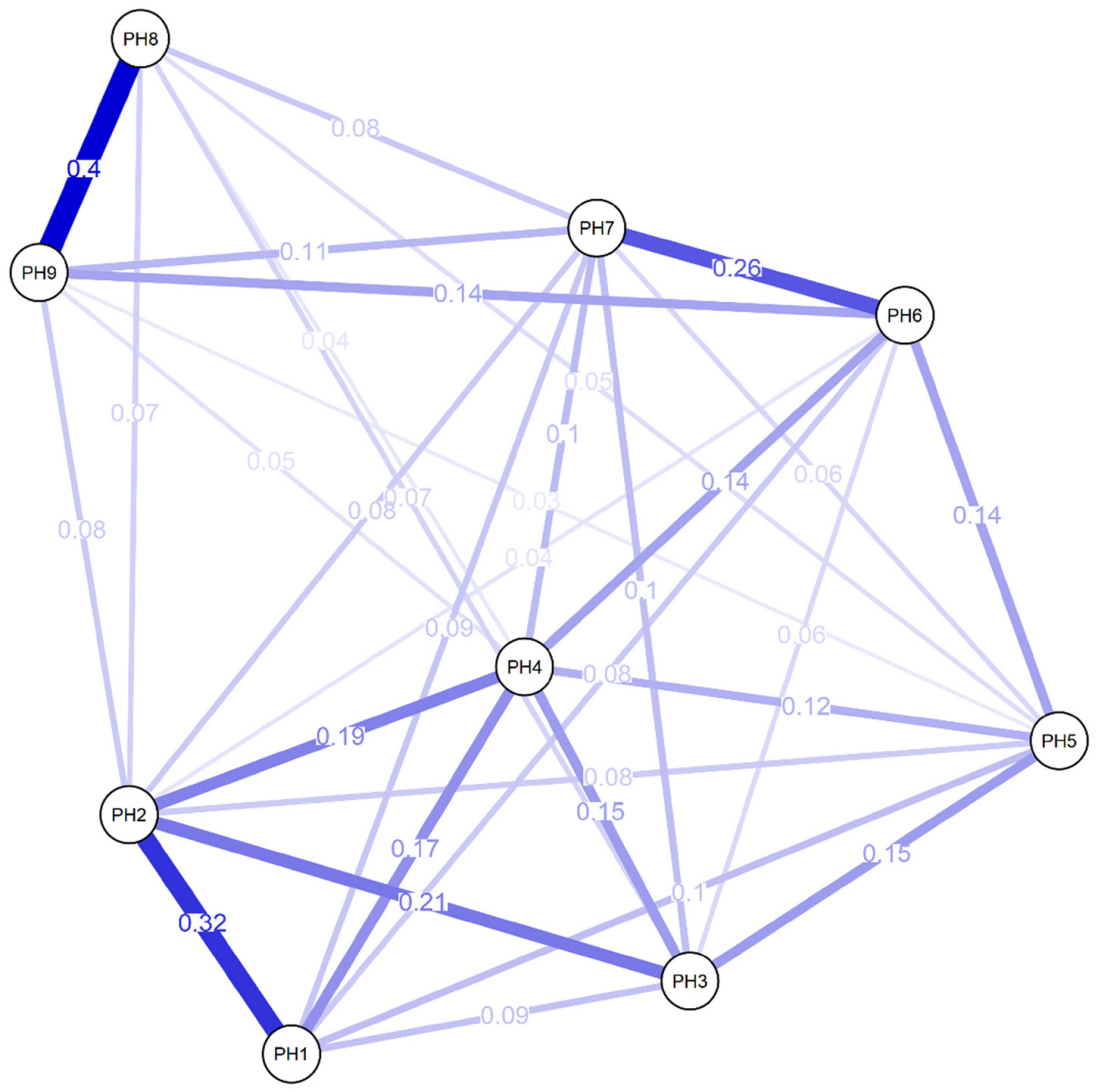
Network analysis of PHQ-9.

**Figure 2.**
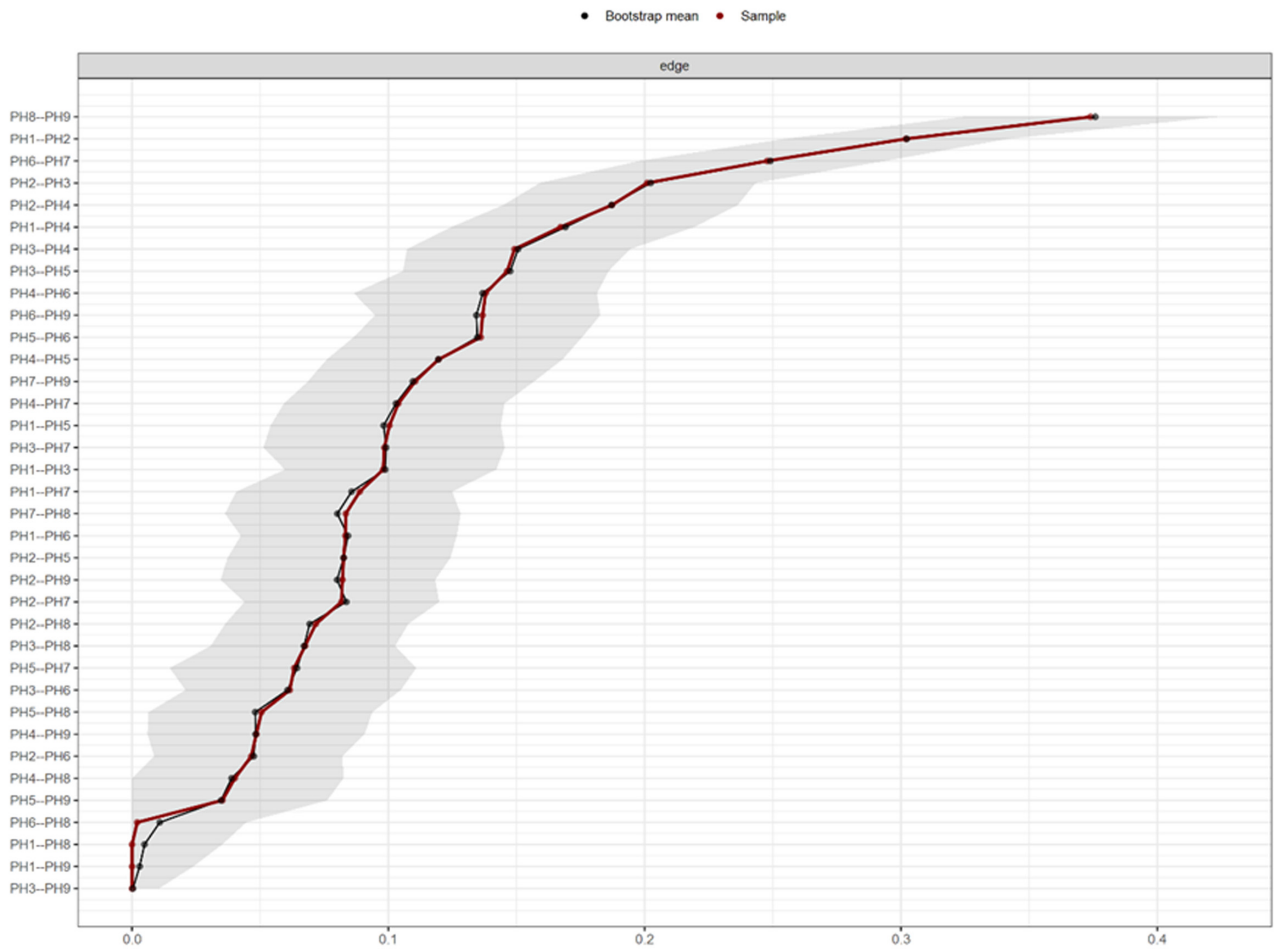
PHQ-9 network estimated border weight confidence intervals.

Figure 1 shows the network chart of PHQ-9 in Peruvian adults with arterial hypertension, where most of the elements are positively associated with a total of 32 possible edges, in which the highest magnitude associations are found with “Moving/ restless” (PH8) and “Suicidal thoughts” (PH9). Also highlighted is the relationships between “Moving/ restless” (PH8) “Interest loss” (PH1) and “Depressed mood” (PH2), and the connection of “Feelings of worthlessness” (PH6) and “Trouble concentrating” (PH7). Other measures of centrality have highlighted greater closeness (1.57) and brokering (1.12) in reagent 6. Figure 2 shows the PHQ-9 network estimated border weight confidence intervals.

Regarding the accuracy of network connection magnitudes calculated by Bootstrap analysis, the analysis indicates that there is a high precision with the dependent intervals of the evaluated network.

## Discussion

This research aimed to explore the dynamics of depression symptoms in adults having Pulmonary arterial hypertension (PAH). This study is the first to use network analysis for PHQ-9 in the Latin American region such as Peru, although several researches have considered network analysis for psychopathological symptoms which may be up to 13 items. Several network trainings on depression symptoms have been evaluated using diverse clinical samples such as chronic pain, depression, bipolar disorder, cancer, but it had been assumed that network analysis has not been performed on Spanish speaking individuals with HTA ^14^.

Therefore, the importance of identifying the deduced components such as chronic pain, depression, bipolar, disorder, and cancer with a greater focus on evaluating would make it possible to strengthen clinical effectiveness for future interventions in patients diagnosed with HTA. This would be considered only to highlight the strength of the node as the main centeredness index due to its stability ^10^. In this sense, the results revealed that the elements with the most centrality are the items of “depressive mood” and “fatigue or energy loss”, this indication may suggest that these symptoms are probably more prevalent in adults diagnosed with HTA. The results are consistent with the McWilliams *et al.*’s ^15^ network study in a sample of patients with chronic pain, which indicated the importance of such symptoms. Other longitudinal network studies have also reinforced the centrality of the “depressed state” symptom ^16, 17^. Furthermore, a recent systematic study of psychopathological networks ^12^ also reported transversal and longitudinal studies of depressive and anxious symptomatology, where the depressive mood symptom has greater network centrality. Therefore, health professionals may consider these symptoms as being in a negative emotion mood, they can also be indicators for resistance to clinical treatment ^18^.

These findings are similar to previous PHQ-9 network studies in cancer patients ^19^, which indicate a greater centrality in reagent 4 (energy loss), which may suggest an indirect relationship of this symptom in people with an irreversible chronic disease.

## Conclusions

In conclusion, the most central reagents in the network (2 and 4) with the most connections report a moderate relationship and are relatively close to the system. These network findings suggest possible routes of greater concentration and dynamism in the process of depressive symptomatology that at a higher level and prevalence, it is more likely to activate the interactive development of the various symptoms of PHQ-9, which may even lead to a depressive episode. Those reagents could have a more significant influence on the components with greater online covariance such as “Psychomotor issues,” “suicidal ideation,” and “anhedonia.” Therefore, the results will contribute to developing personalized treatments aimed at patients with specific depressive symptoms have also been diagnosed with HTA.

However, the research has the following limitations; for example, the study is cross-sectional, which does not allow inference of whether a given node is caused or caused by another node to which it is connected, considering that they are non-directed networks. Another point is the selected small sample of a national survey, which also does not allow for generalization of the results to other patients with physical disorders.

## Data availability

The data referenced by this article are under copyright with the following copyright statement: Copyright: ï¿½ 2021 Ramos-Vera C et al.

Data associated with the article are available under the terms of the Creative Commons Attribution Licence, which permits unrestricted use, distribution, and reproduction in any medium, provided the original data is properly cited. 

### Underlying data

Zenodo: ENDES2019 Dataset with interpretation on depressive symptomatology in Peruvian adults with HTA. http://doi.org/10.5281/zenodo.4384035 ^13^.

This project contains the following underlying data:

- ENDES2019 Dataset With Code Interpretation.xlsx

Data are available under the terms of the Creative Commons Attribution 4.0 International license (CC-BY 4.0).

## Consent

All informed consent was obtained for experimentation with human subjects. All the participation was utterly consensual, unspecified, and voluntary.
