# The network structure of depressive symptomatology in Peruvian adults with arterial hypertension

**DOI:** 10.12688/f1000research.27422.2

**Published:** 2021-03-30

**Authors:** Cristian Ramos-Vera, Jonatan Banos-Chaparro, Roseline Oluwaseun Ogundokun

**Affiliations:** 1 Faculty of Health Sciences, Research Area, Cesar Vallejo University, 640 Del Parque Avenue, San Juan de Lurigancho, 15434, Peru; 2 Norbert Wiener University, Peru, 46970, Peru; 3 Department of Computer Science, Landmark University Omu Aran, Omu Aran, Kwara State, 251101, Nigeria

**Keywords:** Hypertension, Depression, Mental Health, Health Surveys, Peru

## Abstract

**Background:** Globally, arterial hypertension (AH) has increased by 90% over the last four decades, and has increased by 1.6% in Peru over the previous four years. Scientific evidence indicates the prevalence of depressive symptoms in patients with AH and its importance in the comprehensive evaluation of the adult for adherence to clinical treatment. Previous studies carried out in the Peruvian population with AH mostly report the prevalence and associations, but do not indicate which depressive symptoms are more relevant in patients with AH. This study involved a network analysis of depressive symptomatology in Peruvian patients with AH using network estimation. Network analysis is used in this study for analysis, control, and monitoring purposes.

**Method:** A representative cross-sectional study at the national level, using secondary data from 2019 Demographic and Family Health Survey (ENDES) was performed. The sample used included men and women of age over 17 years diagnosed with AH and was able to respond to Patient Health Questionnaire-9 (PHQ-9).

**Results:** The symptoms of depressive mood (bridging force and centrality) and energy fatigue or loss (bridge centrality) play an essential role in the network structure, as does the feeling of uselessness in terms of closeness and intermediation.

**Conclusion:** The study highlighted the symptoms related to depressive mood and energy fatigue or loss as bridging symptoms, which could trigger a depressive episode in patients diagnosed with AH. The results will contribute to developing personalized treatments aimed at patients with specific depressive symptoms who have also been diagnosed with AH. The study analysis presents statistical coefficients of effect size (≤ 0,1 = small; > 0,1 to < 0,5 = moderate; ≥ 0,5 = large) to determine network connections.

## Introduction

Diagnoses of arterial hypertension (AH) among other chronic non-communicable diseases are common ^1^. It necessarily requires a change of lifestyle that favors the adherence to pharmacological and psychological treatments, to reduce the development of cardiovascular diseases or psychological problems which complicate the patient’s health condition. An international study based on various surveys and reviews from 200 countries indicated that AH cases worldwide have increased by 90% over the last four decades, with issues mostly identified in low- and middle-income countries ^2^. In Peru, the prevalence of AH has increased in recent years: 2016 (8.6%), 2017 (8.7%), 2018 (9.5%) and 2019 (10.2%) ^3^. This increase is due to a rise in the population of older people and various lifestyle factors (such as food, for instance, saturated fatty acids, salt sugar, and so on, minimal physical activity, alcohol consumption, among others) ^3^. Systematic review research conducted by Li *et al.*, ^4^ described how common depression is among people with a diagnosis of AH.

Likewise, there is evidence that patients suffering from AH have a higher frequency of presenting emotional disorders, mainly depressive symptoms (40%), anxiety (56%), or stress (20%) ^5^, which interferes with their clinical treatment, leading to poor prognosis (not following the doctor’s instructions regarding medicines, minimal personal care) and preventing the acquisition of desirable behaviors to improve their quality of life ^5^. A recent study in the Peruvian population indicated that depressive symptoms are most likely to occur in the first year of diagnosis of hypertension ^6^. On the other hand, although in chronic disease the development of emotional disorders is likely, there is evidence that emotional disorders contribute to the development of chronic diseases, for example, a study concluded that the greater the depressive symptoms and anxiety, the greater the probability. The fact that the person is diagnosed with AH 5 years later than ^7^ and, on the other hand, a greater number of prescribed medications, is considered a risk factor in the development of depressive symptoms in patients with AH ^8^.

Several studies have indicated that clinical interventions should primarily focus on depression in patients with AH ^5, 7^ since its prevalence is between 3% and 23.7% in the Peruvian adult population ^5, 9^, while international studies report between 4% and 38% ^5, 10^. Patients diagnose with AH normally experience negative emotions because they have to consume the drugs prescribed for the treatment for the rest of their lives or a very long period, these emotions are more powerful in situations where their condition is severe, and may generate feelings of loss of control or fear of failure, thus making it more likely that those with AH condition can develop some emotional disturbances ^11^. These emotions are also related to the economic expenses involved in treatment (especially in low and middle-income countries) and the decrease in social interaction with friends or family ^1^. In this sense, research reveals that health professionals should pay greater attention to negative emotions that occur in patients with AH ^5^ since their evaluation and intervention are important in adherence to clinical treatment. Previous studies carried out in Peru in patients with AH mostly report the prevalence and its association with depressive symptoms ^5, 6, 9^, but they do not indicate which depressive symptom is more relevant or important in patients with AH, which would be appropriate to know this nature. One approach to solving this question is network analysis. This type of analysis makes it possible to analyze how individual behaviors or symptoms are associated with each other and, in turn, how they reinforce and interact. That is, the symptoms and their interaction with other symptoms are the main problem and individual identities, which are not explained by a common latent disorder or cause, mostly explained by the classical medical model ^12^. In this sense, network analysis is relevant to understand and explain psychopathological phenomena, which would allow focusing clinical interventions on specific central symptoms to prevent others, for example, to investigate which depressive symptoms are more important in Peruvian patients diagnosed with AH. Ignoring negative emotions may result in physical disorders ^13^. These are likely to decrease adherence to treatments where psychological support is needed, especially the ones associated with risky behaviors such as alcohol consumption ^5, 14^.

Therefore, the research aimed to explore the network dynamics of depressive symptomatology in Peruvian adults with arterial hypertension from a network analysis approach, which allows a broad understanding of the interactions and the bridges force or centrality between the depressive symptoms and the study population.

## Methods

A secondary cross-sectional study was conducted based on data from ENDES 2019, which is a national representative survey that collects information on chronic non-communicable diseases and gives access to diagnostic and treatment services in Peru. ENDES design includes a two-stage random sampling technique, differentiated for rural and urban areas. In rural areas, the primary sampling units were groups of 500–2000 individuals and the secondary sampling units were the households within each of these groups. On the other hand, in urban areas, the sampling units consisted of blocks or groups of blocks with more than 2,000 individuals and an average of 140 households. The secondary sampling units were the same as in rural settings where we have 36,760 sampled households, 34971 persons aged 15 and older who were surveyed with the Health questionnaire. Details of data sampling, processing, and collection are contained in the ENDES technical report produced by the National Institute of Statistics and Data Processing (INEI).

 Our sample included men and women over the age of 17 diagnosed with AH. The diagnostic criteria for AH were those with systolic blood pressure greater than and/or equal to 140 mmHg and/or diastolic blood pressure greater than and/or equal to 90 mmHg ^15^, and had completed the Patient Health Questionnaire (PHQ-9) ^16^. Exclusion criteria were those who did not meet the diagnostic criteria for hypertension or did not report any blood pressure measurements, or omitted any PHQ-9 questions. This allows evaluation of each of the nine DSM-IV depression criteria. PHQ-9 has four response options (0 = nothing at all, 1 = several days, 2 = more than half of days, and 3 = almost every day) and assesses the presence of depressive symptomatology in the last two weeks, the overall response score is in the range of 0 to 27.

### Sample

A total of 2915 participants were included in this study, of whom 1106 (37.94%) were male, and 1809 (63.06%) were female. The mean age was 57.9 years (standard deviation: 16.9), with 1456 (49.88%) being older adults (55 years old and over). Of these, 1144 (39.24%) had completed primary education, 844 (28.95%) secondary, 637 (21.75%) higher and 290 (9.94%) did not answer. Regarding the participants' native language, 2106 (72.24%) indicated Spanish, 688 (23.6%) Quechua, and 121 (4.16%) a different native language.

### Data analysis

For the analysis of the data, the graph package version 1.6.5 ^17^ was used in the statistical software R version 4.0.3, which allows estimation of a Gaussian chart model (GGM) of a regularized partial correlation network to model the interaction between the components of PHQ-9 as autonomous entities, which are represented as circles, called “nodes”. Nodes are connected by lines, called “edges.” Edges in GGM can be understood as conditional dependency relationships between elements. If two items are connected to the resulting network, they are dependent after all other items are adjusted. This analysis presents statistical coefficients of effect size (≤ 0,1 = small; > 0,1 to < 0,5 = moderate; ≥ 0,5 = large) to determine network connections. The precision of the edge weights was estimated to provide greater stability to the results, with a precision of 95% of the confidence intervals through Bootstrapping of 5000 samples around each edge in the network ^16^. Also considered were the most commonly used centrality indices in psychological networks: strength, closeness, betweenness ^18^. 

### Ethical considerations

The investigation did not require the approval of an ethics committee because it only involved the analysis of secondary data obtained from a public and open-source, which does not require the identification of the participants and maintains the anonymity of the participants.

## Results

In Table 1, the mean scores for each item of the PHQ-9 are shown ^19^. This shows that people with arterial hypertension have a higher level of “Depressed mood”. The item also reports that there is a more significant measure of force in terms of other depressive symptoms. Another essential item on the web was Tired or little energy. The elements of lower centrality were “Moving/restless” and “Appetite change”. Table 2 shows all the PHQ-9 items weight partial correlation matrix.

**Table 1.** Mean and power centrality of items of PHQ-9.

Depression symptoms (PHQ-9)	Mean (ME)	Strength
PH1. Interest loss	0,63	-0,60
PH2. Depressed mood	0,77	1,93
PH3. Trouble sleeping	0,63	-0,25
PH4. Tired or little energy	0,55	0,96
PH5. Appetite change	0,44	-1,06
PH6. Feelings of worthlessness	0,38	0,06
PH7. Trouble concentrating	0,40	0,27
PH8. Moving slowly/restless	0,23	-1,37
PH9. Suicidal thoughts	0,29	-0,47

**Table 2.** Weight matrix of PHQ-9 items.

Network									
Variable	PH1	PH2	PH3	PH4	PH5	PH6	PH7	PH8	PH9
PH1	0.000	0.318	0.095	0.170	0.101	0.082	0.088	0.000	0.000
PH2	0.318	0.000	0.209	0.193	0.081	0.042	0.081	0.071	0.082
PH3	0.095	0.209	0.000	0.152	0.152	0.059	0.100	0.067	0.000
PH4	0.170	0.193	0.152	0.000	0.122	0.142	0.104	0.038	0.046
PH5	0.101	0.081	0.152	0.122	0.000	0.141	0.062	0.051	0.033
PH6	0.082	0.042	0.059	0.142	0.141	0.000	0.261	0.000	0.141
PH7	0.088	0.081	0.100	0.104	0.062	0.261	0.000	0.084	0.112
PH8	0.000	0.071	0.067	0.038	0.051	0.000	0.084	0.000	0.396
PH9	0.000	0.082	0.000	0.046	0.033	0.141	0.112	0.396	0.000

**Figure 1.**
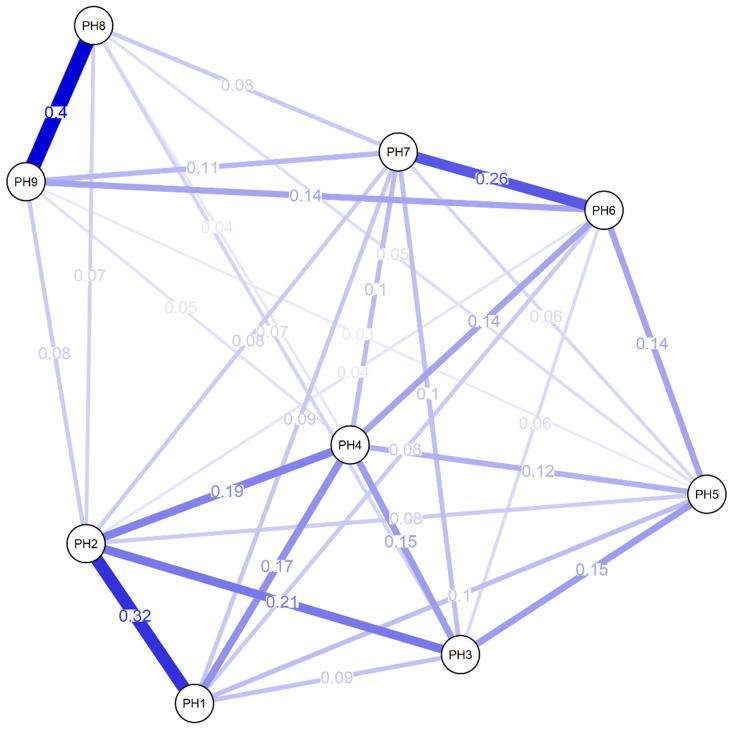
Network analysis of PHQ-9.

**Figure 2.**
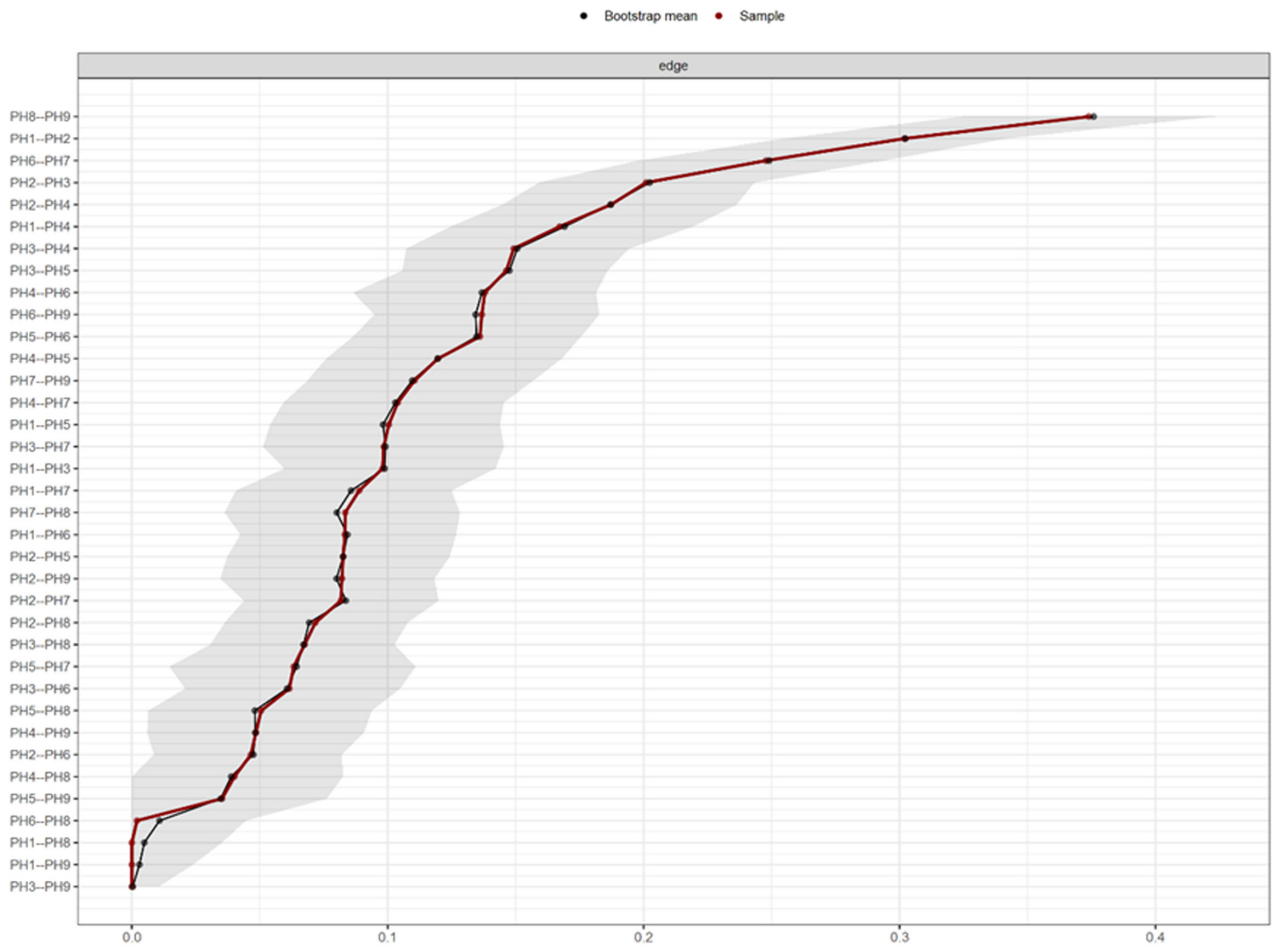
PHQ-9 network estimated border weight confidence intervals.

Figure 1 shows the network chart of PHQ-9 in Peruvian adults with arterial hypertension, where most of the elements are positively associated with a total of 36 possible edges, in which the highest magnitude associations are found with “Moving/ restless” (PH8) and “Suicidal thoughts” (PH9). Also highlighted are the relationships between “Moving/ restless” (PH8) “Interest loss” (PH1) and “Depressed mood” (PH2), and the connection of “Feelings of worthlessness” (PH6) and “Trouble concentrating” (PH7). Other measures of centrality have highlighted greater closeness (1.57) and brokering (1.12) in reagent 6. Figure 2 shows the PHQ-9 network estimated border weight confidence intervals.

Regarding the accuracy of network connection magnitudes calculated by Bootstrap analysis, the analysis indicates that there is high precision with the dependent intervals of the evaluated network.

## Discussion

This research aimed to explore the dynamics of depression symptoms in adults having Pulmonary arterial hypertension (PAH). This study is the first to use network analysis for PHQ-9 in the Latin American region such as Peru, although several kinds of research have considered network analysis for psychopathological symptoms which may be up to 13 items. Several network training on depression symptoms has been evaluated using diverse clinical samples such as chronic pain, depression, bipolar disorder, cancer, but it had been assumed that network analysis has not been performed on Spanish-speaking individuals with AH ^20^.

Bringmann *et al.* ^18^ suggested the utilization of these measures with substantial care thus postulating 3 ways forward. The first is employing a novel centrality measure, the second was to employ and enhance the measures of importance that have been explicitly established for the statistical models which are utilized for the psychological network. The last is leaving the entire idea of centrality entirely behind. Lately, numerous investigators have similarly raised reservations concerning the utilization of centrality indices in psychological networks ^21– 24^. The first limitation is that centrality indices were initially established for social networks which vary from psychological networks in imperative ways. Secondly, centrality indices particularly closeness and betweenness centrality have been demonstrated to be unbalanced in few cross-sectional and temporal networks ^23, 25^. The third is that little study has been conducted on the predictive power of centrality indices ^26^. The researchers concluded that clinicians may perhaps employ extremely central symptoms of cross-sectional networks but merely treating the utmost frequently conveyed symptoms would perhaps exert better ^27^.

Therefore, the importance of identifying the deduced components such as chronic pain, depression, bipolar, disorder, and cancer with a greater focus on evaluating would make it possible to strengthen clinical effectiveness for future interventions in patients diagnosed with AH. This would be considered only to highlight the strength of the node as the main centeredness index due to its stability ^16^. In this sense, the results revealed that the elements with the most centrality are the items of “depressive mood” and “fatigue or energy loss”, this indication may suggest that these symptoms are probably more prevalent in adults diagnosed with AH. The results are consistent with the McWilliams *et al.*’s ^28^ network studies in a sample of patients with chronic pain, which indicated the importance of such symptoms. Other longitudinal network studies have also reinforced the centrality of the “depressed state” symptom ^26, 29^. Furthermore, a recent systematic study of psychopathological networks ^18^ also reported transversal and longitudinal studies of depressive and anxious symptomatology, where the depressive mood symptom has greater network centrality. Therefore, health professionals may consider these symptoms as being in a negative emotional mood, they can also be indicators for resistance to clinical treatment ^30^.

These findings are similar to previous PHQ-9 network studies in cancer patients ^31^, which indicate a greater centrality in reagent 4 (energy loss), which may suggest an indirect relationship of this symptom in people with an irreversible chronic disease.

## Conclusions

In conclusion, the most central reagents in the network (2 and 4) with the most connections report a moderate relationship and are relatively close to the system. These network findings suggest possible routes of greater concentration and dynamism in the process of depressive symptomatology that at a higher level and prevalence, it is more likely to activate the interactive development of the various symptoms of PHQ-9, which may even lead to a depressive episode. Those reagents could have a more significant influence on the components with greater online covariance such as “Psychomotor issues,” “suicidal ideation,” and “anhedonia.” Therefore, the findings of this study will contribute to developing personalized treatments aimed at patients with specific depressive symptoms who have also been diagnosed with AH.

However, the research has the following limitations; for example, the study is cross-sectional, which does not allow inference of whether a given node is caused or caused by another node to which it is connected, considering that they are non-directed networks. Another point is the selected small sample of a national survey, which also does not allow for generalization of the results to other patients with physical disorders. Another limitation is that the sample did not exclude people with other serious diseases that are usually associated with high blood pressure, and which could also be related to the appearance of depressive manifestations (for example stroke or chronic kidney disease).

## Data availability

### Underlying data

Zenodo: ENDES2019 Dataset with interpretation on depressive symptomatology in Peruvian adults with HTA. http://doi.org/10.5281/zenodo.4384035 ^19^.

This project contains the following underlying data:

- ENDES2019 Dataset With Code Interpretation.xlsx

Data are available under the terms of the Creative Commons Attribution 4.0 International license (CC-BY 4.0).

## Consent

All informed consent was obtained for experimentation with human subjects. All the participation was utterly consensual, unspecified, and voluntary.
