# Supplementary material for: The network structure of depressive symptomatology in Peruvian adults with arterial hypertension
Source: F1000Res. 2022 Mar 17;10:19. Originally published 2021 Jan 12. [Version 3] doi: 10.12688/f1000research.27422.3 (PMC9021682; doi:10.12688/f1000research.27422.3)
Supplement: Peer review history

# Peer review: Reviewer response for version 3

**DOI:** 10.5256/f1000research.122354.r127835

**Published:** 2022-04-11

**Authors:** Rahul Shidhaye

**Affiliations:** 1 Pravara Institute of Medical Sciences, Loni, India

Thank you for your response.

Is the work clearly and accurately presented and does it cite the current literature?

Partly

If applicable, is the statistical analysis and its interpretation appropriate?

Partly

Are all the source data underlying the results available to ensure full reproducibility?

Partly

Is the study design appropriate and is the work technically sound?

Partly

Are the conclusions drawn adequately supported by the results?

Partly

Are sufficient details of methods and analysis provided to allow replication by others?

Partly

Reviewer Expertise:

NA

I confirm that I have read this submission and believe that I have an appropriate level of expertise to confirm that it is of an acceptable scientific standard.

# Peer review: Reviewer response for version 2

**DOI:** 10.5256/f1000research.55636.r82483

**Published:** 2021-05-19

**Authors:** Rahul Shidhaye

**Affiliations:** 1 Pravara Institute of Medical Sciences, Loni, India

Thank you so much for your responses. In my understanding the findings of this study are not similar to those reported by McWilliams et al., who have reported that in addition to the depressed mood and tiredness/low energy, two other symptoms - loss of interest and trouble concentrating also have high centrality. This study reports higher centrality only for tiredness/low energy and depressed mood. Please clearly mention this in the discussion.

Is the work clearly and accurately presented and does it cite the current literature?

Partly

If applicable, is the statistical analysis and its interpretation appropriate?

Partly

Are all the source data underlying the results available to ensure full reproducibility?

Partly

Is the study design appropriate and is the work technically sound?

Partly

Are the conclusions drawn adequately supported by the results?

Partly

Are sufficient details of methods and analysis provided to allow replication by others?

Partly

Reviewer Expertise:

NA

I confirm that I have read this submission and believe that I have an appropriate level of expertise to confirm that it is of an acceptable scientific standard, however I have significant reservations, as outlined above.

# Response

**Published:** 2021-11-18

**Authors:** Roseline Ogundokun

**Affiliations:** Landmark University, Nigeria

The results agree with the network study by McWilliams et al. [28] in a sample of patients with chronic pain, which reported 4 symptoms with the highest centrality, where depressed mood and fatigue or loss of tiredness had the highest values. high. However, the authors indicate the relevance of two other symptoms, for example, loss of interest and concentration difficulties. These last two symptoms were not found in our study and a probable explanation is due to the type of population. The authors indicate that people who experience chronic pain are also less able to devote their attention to other activities or simply lose interest since it is difficult for them to concentrate while in pain28. For this reason, in patients with chronic pain, it is even more likely to find more central symptoms due to the persistent pain that generates discomfort, compared to patients with HA who do not present this type of chronic pain.

# Response

**Published:** 2022-03-10

**Authors:** Roseline Ogundokun

**Affiliations:** Landmark University, Nigeria

This is the response to your concern and it has been added in the discussion section as suggested by you. 

 "The results agree with the network study by McWilliams et al. [28] in a sample of patients with chronic pain, which reported 4 symptoms with the highest centrality, where depressed mood and fatigue or loss of tiredness had the highest values. high. However, the authors indicate the relevance of two other symptoms, for example, loss of interest and concentration difficulties. These last two symptoms were not found in our study and a probable explanation is due to the type of population. The authors indicate that people who experience chronic pain are also less able to devote their attention to other activities or simply lose interest since it is difficult for them to concentrate while in pain [28]. For this reason, in patients with chronic pain, it is even more likely to find more central symptoms due to the persistent pain that generates discomfort, compared to patients with HA who do not present this type of chronic pain".

# Peer review: Reviewer response for version 2

**DOI:** 10.5256/f1000research.55636.r82482

**Published:** 2021-04-09

**Authors:** Santiago Stucchi-Portocarrero

**Affiliations:** 1 Hospital Víctor Larco Herrera, Universidad Peruana Cayetano Heredia, Magdalena del Mar, Peru

I think that "over the age of 17" is not very clear. Maybe it would be better "18 years and older".

 The authors still mentioned “Pulmonary arterial hypertension (PAH)” in the discussion, although it is not mentioned in the rest of the article.

 Finally, I still believe that the authors should better explain how the results of the study can lead to personalized treatments.

Is the work clearly and accurately presented and does it cite the current literature?

Partly

If applicable, is the statistical analysis and its interpretation appropriate?

I cannot comment. A qualified statistician is required.

Are all the source data underlying the results available to ensure full reproducibility?

Yes

Is the study design appropriate and is the work technically sound?

Yes

Are the conclusions drawn adequately supported by the results?

Partly

Are sufficient details of methods and analysis provided to allow replication by others?

Yes

Reviewer Expertise:

Clinical psychiatry, suicide, psychopharmacology, history of psychiatry

I confirm that I have read this submission and believe that I have an appropriate level of expertise to confirm that it is of an acceptable scientific standard.

# Peer review: Reviewer response for version 1

**DOI:** 10.5256/f1000research.30303.r78628

**Published:** 2021-03-01

**Authors:** Rahul Shidhaye, Rachana Parikh

**Affiliations:** 1 Pravara Institute of Medical Sciences, Loni, India; 2 PATH, New Delhi, Delhi, India

In this paper, Cristian Ramos-Vera and colleagues present the findings of network analysis of symptoms of depression in adults with arterial hypertension in Peru. This is a secondary data analysis of a nationally representative survey: ENDES 2019. Very few studies have applied network analysis in the field of mood disorders, especially for co-morbid depression ^1^. This is quite a unique study for which we would like to congratulate the authors. However, there are some issues related to methodology, presentation of results, and discussion of limitations which need to be addressed by the authors.

Abstract

1. Background: The first word should be edited to “Globally, arterial hypertension…” instead of “Global arterial hypertension…”. The salience of depression as a comorbidity among people with arterial hypertension needs to be highlighted. Please mention the rationale for using network analysis.
2. Methods: The authors need to clarify the sample included in the study, particularly that: 1) it is a sub-sample (people with arterial hypertension) from the ENDES 2019 dataset, 2) clarify if only those who had ‘complete PHQ-9 questionnaires’ were included.
3. Results: The authors have used terms such as bridging force/ bridge centrality, closeness, and intermediation in the abstract, which are different from the corresponding terms in the manuscript. The authors need to use uniform and standard terminology throughout the paper. These results need to also include the corresponding coefficients for the results.
4. Conclusion: The conclusion reads like results. The interpretation of the results and its implications should be included.

Introduction

1. The authors need to describe how common is depression among people with a diagnosis of arterial hypertension in this section.
2. The appropriate abbreviation for Arterial Hypertension would be AHT or AH. It is not clear why HTA is used.
3. Referring to the sentence - “This increase is due to… such as food” – the type of food needs to be included to suggest poor eating habits.
  0
4. Referring to the sentence – “There is also evidence that patients with HTA have a higher incidence of emotional disorders” – please include how much higher and include appropriate reference.
5. In the same sentence “… mainly depressive symptomatology (anxiety or stress)” – the use of parenthesis is confusing. Perhaps the authors mean “…depressive symptomatology, anxiety and stress”.
6. The sentence “Ignoring negative emotions may result in physical disorders.” – This requires appropriate citation and some further explanation.
7. The rationale for undertaking network analysis and the need to explore network dynamics for symptoms of depression in individuals with hypertension needs to be clearly explained.
8. In the last paragraph of the introduction, the terms used are ‘interactions’ and ‘bridges of connections,’ which are different than the terms used in the abstract earlier and the methods section later (refer to comment # 3 in the section on abstract). As this is a new field, it is important to clearly define the key terms preferably using a box.

Methods

1. Brief information about ENDES dataset, including the disorders screened/diagnosed for the sample, particularly how mental health problems were screened/diagnosed need to be described. Please provide the reference for this study as well.  
2. The sentence describing the sampling, “On the other hand, in urban areas… with the Health questionnaire” is confusing. Perhaps a full stop was meant to be included before “from 36,760 sampled households,”.
3. The authors might have missed to add “did not” in the sentence,” Exclusion criteria were those who (did not) me(e)t the diagnostic criteria for hypertension, or did not report any blood pressure measurements, or who omitted any PHQ-9 questions.
4. Please provide the flow chart for sample selection. It is particularly important to provide the details regarding the differences between those included and excluded in the analysis. The details related to socio-demographic indicators, severity of hypertension and PHQ-9 scores should at least be included.
5. Missing data should be reported, and especially how it was handled should be described, along with how this is likely to influence the results, if at all. 
6. The commonly used term to describe the connection between nodes is ‘edges.’ ^1^. It is not clear why the authors have used the term ‘border.’
7. Bringmann et al. have used the terms degree and strength centrality, betweenness and closeness centrality to describe centrality indices. Authors have cited this paper (reference # 12) ^2^. Please clarify why the terms force, proximity, and intermediation are used by the authors while they cite the above-mentioned paper.

Results

1. For a network with 9 nodes, the possible number of edges is (n*(n-1))/2, i.e., (9*8)/2=36. Not clear why it is mentioned ‘32 possible edges.’
2. It is desirable to have a table which includes PHQ-9 scores for the sample, mean with standard deviation, and proportion of sample that is moderately/severely depressed. This could be disaggregated by age, sex, hypertension, socio-economic status and other independent variables.
3. Please provide partial correlation matrix for all PHQ-9 items.
4. The findings related to only strength centrality are presented in table 1. Please also include the findings pertaining to betweenness and closeness centrality.
5. Please clarify the meaning of negative sign in strength column.
6. Please consider presenting the findings separately for those without depression and those with depression (PHQ-9>5). Similarly present the findings disaggregated by sex and severity of depression.
7. Please explain non-inclusion of socio-demographic factors and hypertension in the network analysis. Factors others than the symptoms of depression are included in the analysis to see how they influence each other.
8. Is it possible to present predictability analysis as suggested by Contreras et al. ^1^

Discussion and Conclusion

1. Greater centrality for PHQ-6 needs to be further discussed. PHQ-8 and PHQ-9 are clinically very significant but have low centrality measures. This also needs to be discussed.
2. It will be good to discuss the findings related to various centrality indices in detail.
3. The discussion could benefit from including more critical analysis of how their findings differ from other studies and the implications, therefore. For e.g., McWilliams et al (ref 15 in the paper) found that in addition to the depressed mood and tiredness/low energy, two other symptoms - loss of interest and trouble concentrating also have high centrality, which is in contrast with the authors findings. The discussion could explore potential reasons for this and implications.
4. The first paragraph of conclusion appears to fit better with the discussion section.
5. The authors should elaborate and include reference for the statement, “Those reagents could have a more significant influence on the components with greater online covariance such as “Psychomotor issues,” “suicidal ideation,” and “anhedonia.”
6. Referring to sentence, “Therefore, the results will contribute to… have also been diagnosed with HTA.” This statement needs to re-phrased for better readability.
7. There are several issues related to use to centrality indices and are discussed in detail by Bringmann et al. They have recommended to use these measures with considerable care. Authors need to include a paragraph to discuss this and highlight the limitation of using centrality indices.
8. It will be desirable to provide R Script as an appendix. This will ensure the reproducibility of the results.

Is the work clearly and accurately presented and does it cite the current literature?

Partly

If applicable, is the statistical analysis and its interpretation appropriate?

Partly

Are all the source data underlying the results available to ensure full reproducibility?

Partly

Is the study design appropriate and is the work technically sound?

Partly

Are the conclusions drawn adequately supported by the results?

Partly

Are sufficient details of methods and analysis provided to allow replication by others?

Partly

Reviewer Expertise:

Rahul Shidhaye: Epidemiology, public mental health, depression, health services research  Rachana Parikh: Global Mental Health, Adolescent Health, Epidemiology, Health Systems research.

We confirm that we have read this submission and believe that we have an appropriate level of expertise to confirm that it is of an acceptable scientific standard, however we have significant reservations, as outlined above.

## References

[1] : The Study of Psychopathology from the Network Analysis Perspective: A Systematic Review. *Psychother Psychosom* .2019;88(2) : 10.1159/000497425 71-83 10.1159/000497425 30889609

[2] : What do centrality measures measure in psychological networks?. *J Abnorm Psychol* .2019;128(8) : 10.1037/abn0000446 892-903 10.1037/abn0000446 31318245

# Response

**Published:** 2021-03-22

**Authors:** Roseline Ogundokun

**Affiliations:** Landmark University, Nigeria

**Title**. The authors describe the population studied as “Peruvian adults”. However, the sample used included 17-year-olds, and in Peru only those over 18 are considered adults.

**Response: It was stated in the article that more than 17years of age which means from 18years and above**

**Introduction**. “HTA” is the acronym for *hipertensión arterial *(arterial hypertension in Spanish); it is not applicable in an article published in English. On the other hand, the relationship between depression and arterial hypertension is described; however, it is necessary to analyze other possibilities. For example, depression and chronic anxiety are considered risk factors for the development of high blood pressure, and emotional disturbances might be the adverse effect of some antihypertensive medications.

**Response: The comment has been attended to.**

**Methods**. It should be mentioned that there is a validation of PHQ-9 for the Peruvian population, which was done precisely from ENDES 2019 1 Also, there is an obvious error in the following sentence: "Exclusion criteria were those who met the diagnostic criteria for hypertension."

**Response: The comment has been attended to.**

**Discussion.** The authors mentioned that “this research aimed to explore the dynamics of depression symptoms in adults having Pulmonary arterial hypertension (PAH).” However, in the rest of the article only arterial hypertension is mentioned, without further specification.

**Response: The comment has been attended to.**

**Conclusions**. It should be mentioned, as a limitation, that the sample did not exclude people with other serious diseases that are usually associated with high blood pressure, and which could also be related to the appearance of depressive manifestations (for example: stroke or chronic kidney disease). Finally, it is not clear how the results of the study can lead to personalized treatments; this part should be better explained by the authors.

**Response: The comment has been attended to.**

# Peer review: Reviewer response for version 1

**DOI:** 10.5256/f1000research.30303.r78631

**Published:** 2021-02-12

**Authors:** Santiago Stucchi-Portocarrero

**Affiliations:** 1 Hospital Víctor Larco Herrera, Universidad Peruana Cayetano Heredia, Magdalena del Mar, Peru

**Title**. The authors describe the population studied as “Peruvian adults”. However, the sample used included 17 year olds, and in Peru only those over 18 are considered adults.

**Introduction**. “HTA” is the acronym for *hipertensión arterial* (arterial hypertension in Spanish); it is not applicable in an article published in English. On the other hand, the relationship between depression and arterial hypertension is described; however, it is necessary to analyze other possibilities. For example, depression and chronic anxiety are considered risk factors for the development of high blood pressure, and emotional disturbances might be the adverse effect of some antihypertensive medications.

**Methods**. It should be mentioned that there is a validation of PHQ-9 for the Peruvian population, which was done precisely from ENDES 2019 ^1^ Also, there is an obvious error in the following sentence: "Exclusion criteria were those who met the diagnostic criteria for hypertension."

 Discussion. The authors mentioned that “this research aimed to explore the dynamics of depression symptoms in adults having Pulmonary arterial hypertension (PAH).” However, in the rest of the article only arterial hypertension is mentioned, without further specification.

**Conclusions**. It should be mentioned, as a limitation, that the sample did not exclude people with other serious diseases that are usually associated with high blood pressure, and which could also be related to the appearance of depressive manifestations (for example: stroke or chronic kidney disease). Finally, it is not clear how the results of the study can lead to personalized treatments; this part should be better explained by the authors.

Is the work clearly and accurately presented and does it cite the current literature?

Partly

If applicable, is the statistical analysis and its interpretation appropriate?

I cannot comment. A qualified statistician is required.

Are all the source data underlying the results available to ensure full reproducibility?

Yes

Is the study design appropriate and is the work technically sound?

Yes

Are the conclusions drawn adequately supported by the results?

Partly

Are sufficient details of methods and analysis provided to allow replication by others?

Yes

Reviewer Expertise:

Clinical psychiatry, suicide, psychopharmacology, history of psychiatry.

I confirm that I have read this submission and believe that I have an appropriate level of expertise to confirm that it is of an acceptable scientific standard, however I have significant reservations, as outlined above.

## References

[1] : Valid group comparisons can be made with the Patient Health Questionnaire (PHQ-9): A measurement invariance study across groups by demographic characteristics. *PLoS One* .2019;14(9) : 10.1371/journal.pone.0221717 e0221717 10.1371/journal.pone.0221717 31498796PMC6733536

# Response

**Published:** 2021-03-22

**Authors:** Roseline Ogundokun

**Affiliations:** Landmark University, Nigeria

**Abstract**

 Background: The first word should be edited to “Globally, arterial hypertension…” instead of “Global arterial hypertension…”. The salience of depression as a comorbidity among people with arterial hypertension needs to be highlighted. Please mention the rationale for using network analysis.

**Response: The comment has been attended to**

 Methods: The authors need to clarify the sample included in the study, particularly that: it is a sub-sample (people with arterial hypertension) from the ENDES 2019 dataset.

**Response: This has been discussed in the article**

 clarify if only those who had ‘complete PHQ-9 questionnaires’ were included.

**Response: This has been discussed in the article**

**Response: The comment has been attended to**

 Results: The authors have used terms such as bridging force/ bridge centrality, closeness, and intermediation in the abstract, which are different from the corresponding terms in the manuscript. The authors need to use uniform and standard terminology throughout the paper. These results need to also include the corresponding coefficients for the results.

**Response: The comment has been attended to**

 Conclusion: The conclusion reads like results. The interpretation of the results and its implications should be included.

**Response: The comment has been attended to**

**Introduction**

 The authors need to describe how common is depression among people with a diagnosis of arterial hypertension in this section.

**Response: The comment has been attended to**

 The appropriate abbreviation for Arterial Hypertension would be AHT or AH. It is not clear why HTA is used.

**Response: The comment has been attended to**

 Referring to the sentence - “This increase is due to… such as food” – the type of food needs to be included to suggest poor eating habits.

**Response: The comment has been attended to**

 Referring to the sentence – “There is also evidence that patients with HTA have a higher incidence of emotional disorders” – please include how much higher and include appropriate reference.

**Response: The comment has been attended to**

 In the same sentence “… mainly depressive symptomatology (anxiety or stress)” – the use of parenthesis is confusing. Perhaps the authors mean “…depressive symptomatology, anxiety and stress”.

**Response: The comment has been attended to**

 The sentence “Ignoring negative emotions may result in physical disorders.” – This requires appropriate citation and some further explanation.

**Response: The comment has been attended to**

 The rationale for undertaking network analysis and the need to explore network dynamics for symptoms of depression in individuals with hypertension needs to be clearly explained.

**Response: The comment has been attended to**

 In the last paragraph of the introduction, the terms used are ‘interactions’ and ‘bridges of connections,’ which are different than the terms used in the abstract earlier and the methods section later (refer to comment # 3 in the section on abstract). As this is a new field, it is important to clearly define the key terms preferably using a box.

**Response: The comment has been attended to**

**Methods**

 Brief information about ENDES dataset, including the disorders screened/diagnosed for the sample, particularly how mental health problems were screened/diagnosed need to be described. Please provide the reference for this study as well.

**Response: The comment has been attended to. This has been described in the article and no mental health diagnosis was made. Only those who completed all the PHQ-9 were included.**

 The sentence describing the sampling, “On the other hand, in urban areas… with the Health questionnaire” is confusing. Perhaps a full stop was meant to be included before “from 36,760 sampled households,”.

**Response: The comment has been attended to**

 The authors might have missed to add “did not” in the sentence,” Exclusion criteria were those who (did not) me(e)t the diagnostic criteria for hypertension, or did not report any blood pressure measurements, or who omitted any PHQ-9 questions.

**Response: The comment has been attended to**

 Please provide the flow chart for sample selection. It is particularly important to provide the details regarding the differences between those included and excluded in the analysis. The details related to socio-demographic indicators, severity of hypertension and PHQ-9 scores should at least be included.

**Response: This Has Nothing to do with the study objective and reference was already made to the inclusion and exclusion factors**

 Missing data should be reported, and especially how it was handled should be described, along with how this is likely to influence the results, if at all. The commonly used term to describe the connection between nodes is ‘edges.’1. It is not clear why the authors have used the term ‘border.’

**Response: The missing data did not influence the results, because they were only not reported in relation to the language and grade of studies, whose variables are not in the network analysis.**

 Bringmann et al. have used the terms degree and strength centrality, betweenness and closeness centrality to describe centrality indices. Authors have cited this paper (reference # 12)2. Please clarify why the terms force, proximity, and intermediation are used by the authors while they cite the above-mentioned paper.

**Response: The comment has been attended to**

**Results**

 For a network with 9 nodes, the possible number of edges is (n*(n-1))/2, i.e., (9*8)/2=36. Not clear why it is mentioned ‘32 possible edges.’

**Response: The comment has been attended to. This has been corrected to 36 nodes**

 It is desirable to have a table which includes PHQ-9 scores for the sample, mean with standard deviation, and proportion of sample that is moderately/severely depressed. This could be disaggregated by age, sex, hypertension, socio-economic status and other independent variables.

**Response: The analysis does not have sociodemographic purposes for which it is not considered**

 Please provide partial correlation matrix for all PHQ-9 items.

**Response: This is provided in the article already**

 The findings related to only strength centrality are presented in table 1. Please also include the findings pertaining to betweenness and closeness centrality.

**Response: Reference was made on the use of the only measure that reports greater stability, the others do not allow accurate interpretations, especially with high sample data**

 Please clarify the meaning of negative sign in strength column.

**Response: refers to a negative force**

 Please consider presenting the findings separately for those without depression and those with depression (PHQ-9>5). Similarly present the findings disaggregated by sex and severity of depression.

**Response: The purpose was to evaluate according to the total sample, performing by different categoric variables generates a complexity of different results that do not support the study objective, which, according to the sample size, allows generalizing the symptomatology hypersensitive positivity in priority.**

 Please explain non-inclusion of socio-demographic factors and hypertension in the network analysis. Factor’s others than the symptoms of depression are included in the analysis to see how they influence each other.

**Response: The comment has been attended to**

 Is it possible to present predictability analysis as suggested by Contreras et al.1

**Response: This is not possible because it requires Longitudinal Data**

**Discussion and Conclusion**

 Greater centrality for PHQ-6 needs to be further discussed. PHQ-8 and PHQ-9 are clinically very significant but have low centrality measures. This also needs to be discussed.

**Response: The comment has been attended to.**

 It will be good to discuss the findings related to various centrality indices in detail.

**Response: This was already specified the inclusion of the centrality measures of greatest implication in psychological networks, which must be considered the centrality of force because of its stability.**

 The discussion could benefit from including more critical analysis of how their findings differ from other studies and the implications, therefore. For e.g., McWilliams et al (ref 15 in the paper) found that in addition to the depressed mood and tiredness/low energy, two other symptoms - loss of interest and trouble concentrating also have high centrality, which is in contrast with the authors findings. The discussion could explore potential reasons for this and implications.

**Response: The comment has been attended to.**

 The first paragraph of conclusion appears to fit better with the discussion section.

**Response: The comment has been attended to.**

 The authors should elaborate and include reference for the statement, “Those reagents could have a more significant influence on the components with greater online covariance such as “Psychomotor issues,” “suicidal ideation,” and “anhedonia.”

**Response: The comment has been attended to.**

 Referring to sentence, “Therefore, the results will contribute to… have also been diagnosedwith HTA.” This statement needs to re-phrased for better readability.

**Response: The comment has been attended to.**

 There are several issues related to use to centrality indices and are discussed in detail by Bringmann et al. They have recommended to use these measures with considerable care. Authors need to include a paragraph to discuss this and highlight the limitation of using centrality indices.

**Response: This is already explained in the article**

 It will be desirable to provide R Script as an appendix. This will ensure the reproducibility of the results.

**Response: It is not possible because it is part of another study in progress**
